# Seroprevalence of Herpes Simplex Virus type-2 (HSV-2) among pregnant women who participated in a national HIV surveillance activity in Haiti

**DOI:** 10.1186/s12879-017-2674-4

**Published:** 2017-08-18

**Authors:** Jean Wysler Domercant, Frantz Jean Louis, Erin Hulland, Mark Griswold, Jocelyne Andre-Alboth, Tun Ye, Barbara J. Marston

**Affiliations:** 1 Centers for Disease Control and Prevention, Tabarre, Haiti; 2 Centers for Disease Control and Prevention, 1600 Clifton Rd, Atlanta, GA 30329 USA; 3 National Alliance of State and Territorial AIDS Directors, Washington, USA; 4 National Public Health Laboratory/Ministry of Health of Haiti, Delmas, Haiti

**Keywords:** HSV-2, HIV women, Pregnant women, Prevalence, Screening

## Abstract

**Background:**

Herpes simplex virus type 2 (HSV-2), one the most common causes of genital ulcers, appears to increase both the risk of HIV acquisition and HIV transmission. HSV-2/HIV co-infection among pregnant women may increase the risk of perinatal transmission of HIV. This study describes rates of HSV-2 among pregnant women in Haiti and HSV-2 test performance in this population.

**Methods:**

Unlinked residual serum specimens from the 2012 National HIV and Syphilis Sentinel Surveillance Survey among pregnant women in Haiti were tested using two commercial kits (Focus HerpeSelect, Kalon) for HSV-2 antibodies. We evaluated rates of HSV-2 seropositivity and HSV-2/HIV co-infection, associations between HSV-2 and demographic characteristics using multivariable Cox proportional hazards modeling, and HSV-2 test performance in this population.

**Results:**

Serum samples from 1000 pregnant women (all 164 HIV positive and 836 random HIV negative) were selected. The overall weighted prevalence of HSV-2 was 31.4% (95% CI: 27.7–35.4) and the prevalence of HIV-positivity among HSV-2 positive pregnant women was five times higher than the prevalence among HSV-2 negative women (4.8% [95% CI: 3.9–6.0] vs. 0.9% [95% CI: 0.6–1.3], respectively). Factors significantly associated with HSV-2 positivity were HIV-positivity (PR: 2.27 [95% CI: 1.94–2.65]) and older age (PRs: 1.41 [95% CI: 1.05–1.91] for 20–24 years, 1.71 [95% CI:1.13–2.60] for 30–34 years, and 1.55 [95% CI: 1.10–2.19] for 35 years or greater]), while rural residence was negatively associated with HSV-2 positivity (PR 0.83 [95% CI: 0.69–1.00]), after controlling for other covariables. For this study a conservative Focus index cutoff of 3.5 was used, but among samples with a Focus index value ≥2.5, 98.4% had positive Kalon tests.

**Conclusion:**

The prevalence of HSV-2 is relatively high among pregnant women in Haiti. Public health interventions to increase access to HSV-2 screening in antenatal services are warranted.

## Background

Herpes simplex virus type 2 (HSV-2), the primary cause of genital herpes, is one the most common causes of genital ulcers [1, 2]. HSV-2 can be contracted during pregnancy and can be transmitted to the fetus or newborn, sometimes with devastating consequences [3]. Mortality from neonatal transmission of genital herpes, even in developed countries, may reach 60% and survivors are at risk of major disabilities [3].

Sexually transmitted infections that cause inflammation or ulcerative genital lesions have long been identified as among the greatest risk factors for HIV infection [1, 4], and infection with HSV-2 has been strongly associated with HIV infection [2, 4, 5]. HSV-2 appears to increase both the risk of HIV acquisition and the potential for a co-infected individual to transmit HIV to sexual partners [2, 5]. Moreover, co-infection with HSV-2 and HIV among pregnant women may increase the risk of intrapartum transmission of HIV by as much as 25% [6], particularly among women shedding HSV-2 [7]. The mechanisms through which HSV-2 infection increases the risk of HIV transmission and acquisition are complex; infection with HSV-2 increases the HIV viral load, both in the plasma and in genital secretions [8, 9] and disruption of the mucosa and activation of local populations of T-cells both appear to contribute to the risk of HIV transmission and acquisition [10]. Data from sub-Saharan Africa suggest that the proportion of HIV transmission attributable to HSV-2 infection can be substantial, and may increase with maturity of an HIV epidemic [11]. Since most HSV-2 infections are acquired sexually, rates of HSV-2 seropositivity can provide information about sexual activity at the population level and risk of HIV at the individual level [12].

HSV-2 is widely dispersed globally. Prevalence of HSV-2 among women can vary by region, from 17% in USA [13] to as high as 80% in Sub-Saharan Africa [14]. In general, rates of HSV-2 are higher among women versus men [15] and among pregnant women versus non-pregnant women [16]. Treatment of HSV-2 during pregnancy can reduce the risk of vertical transmission of HSV-2, and for women with prodromal symptoms or active HSV-2 ulceration at the time of labor, cesarean sections can also reduce the transmission risk [17]. Efforts to reduce HIV transmission and acquisition risk through treatment of HSV-2 have been disappointing, but high-dose antivirals (e.g., valacyclovir) may significantly reduce plasma HIV viral level among persons with HSV-2/HIV co-infection and potentially HIV infectiousness [18], and thus strategies to control HSV-2 and its impact on HIV epidemics continue to be evaluated.

Because HSV-2 infection is frequently asymptomatic or under-recognized, serology has been used to evaluate HSV-2 infection rates. Currently available serologic tests for HSV-2 are able to distinguish between HSV types and perform well in some populations; however high rates of false positive tests have been reported in some African populations, possibly related to cross-reactive immunity to other antigens and evaluation of test performance in populations of interest has been recommended [19]. A meta-analysis of results of testing in African populations using two of the most commonly used commercial kits (Focus HerpeSelect, HSV-2 enzyme-linked immunosorbent assay [ELISA], [Focus Technologies, Cypress, CA] and Kalon gG2 ELISA [Kalon Biologicals Ltd., Guilford, United Kingdom]) has been recently reported [19]. The reported summary sensitivity for the Focus kit was 99%. The reported specificity using the manufacturer’s suggested cutoff of 1.1 was only 69%, but specificity improved to 85% (79–92%) when cutoffs of 2.2–3.5 were used.

There are limited data available on HSV-2 prevalence or test performance and absolutely no data on HSV-1 genital herpes in Haiti [20], and there have not been previous studies of the prevalence of HSV-2 among pregnant women. In a survey of commercial sex workers (CSW) and their clients in the Artibonite Department in 2008, 46.7% of female CSW and 22% of their clients were seropositive for HSV-2 [20]. This study reported the results of three serologic tests for HSV-2 (HerpesSelect, Kalon and Capita) and defined overall results as positive when two of three tests yielded positive results.

The availability of banked sera from a survey of HIV and syphilis at sentinel antenatal care (ANC) clinics in Haiti provided an opportunity to evaluate HSV-2 test performance, rates of HSV-2, and the association between HSV-2 and HIV among pregnant women in Haiti.

## Methods

### Study design

We tested a subset of 1000 unlinked residual serum specimens collected as part of the National HIV and Syphilis Sentinel Surveillance Survey among pregnant women in Haiti in 2012 [21]. Eighteen sites, including sites from each of Haiti’s ten administrative departments, were included in the original survey. Individual pregnant women at the sites were included if they were aged 15–49 years, had come for a first prenatal visit (regardless of the stage of pregnancy), and had been offered services related to prevention of mother to child transmission (PMTCT) of HIV, including HIV testing. Selection criteria and non-response are described in full detail in the main 2012 survey [21]. A total of 7077 de-identified residual serum samples were collected, along with limited demographic information. Following testing for HIV and syphilis as part of the original survey, samples were stored at the National Public Health Laboratory (French acronym LNSP) at -80 °C.

### Sample size considerations and selection of sera

From the 7077 serum samples, a subset of 1000 samples were selected for HSV-2 testing, including all sera for which HIV testing was positive (164) and a random sample of 836 of the HIV-negative sera. Samples were tested for HSV-2 if: (i) the sample volume was adequate (minimum 0.5 mL); (ii) there was no evidence that sample integrity was compromised (e.g. evidence of contamination); and (iii) the study number was clearly identified.

### Laboratory

Laboratory testing was conducted at LNSP. Samples were tested for the presence of HSV-2 antibody using the HerpeSelect® ELISA serological testing as per the manufacturer instructions. (Focus HerpeSelect HSV-2 enzyme-linked immunosorbent assay [ELISA] [Focus Technologies, Cypress, CA]).

The manufacturer’s instructions for this assay defined a negative result as an index value less than 0.9, an indeterminate result as an index value between 0.9 and 1.1, and a positive result as an index value greater than 1.1. However because of reports of significant rates of false positivity with the Focus test in some populations using this definition, we chose a conservative definition of a positive test for the purpose of reporting an overall rate of HSV-2 seropositivity as recommended by others [22, 23]; our a priori definition of a positive test for this purpose was a Focus result of ≥3.5 [22, 24].

Samples with index values >1.1 by Focus-HSV-2 ELISA were tested using Kalon HSV-2 gG2 ELISA (Kalon Biologicals). Samples with index values ≥3.5 that were confirmed by Kalon as positive were considered HSV-2 positive for the purposes of epidemiologic analyses.

### Data analysis

We analysed the proportions of the participant population that were seropositive for HSV-2 and for HSV-2/HIV co-infection, accounting for unequal probabilities of selection and department-level clustering using the complex survey procedures in SAS software Version 9.3 –(surveyfreq, surveymeans, surveyreg, surveyphreg) (Cary, NC). Weights were calculated based on the probability of selection into the 1000 serum samples assessed in this analysis.

*P*-values were generated to assess statistical significance between HSV-2 negative and HSV-2 positive demographics via Rao-Scott Chi-Square test for categorical outcomes and via t-tests for continuous variables. Prevalence ratios (PR) were estimated using univariable and multivariable Cox proportional hazards models with equal time to follow-up for all participants; the multivariable model included demographic characteristics and HIV status as covariables. Observations with missing data were excluded from analysis.

We further evaluated the effect of index value cut-off for the HerpeSelect test on test specificity by analyzing the proportion of samples with index values >1.1 that were confirmed by Kalon, stratified into ranges for the index values.

The use and testing of the specimens for this study was approved by the National Bioethics Committee in Haiti, and the study was approved as a non-research evaluation by the U.S. Centers for Disease Control and Prevention.

## Results

Of the 1000 total samples selected, HSV-2 testing was not done for 72 (7.2%), primarily because of insufficient sample volume (*n* = 55, 76.4%). Among the 928 women tested, 31.4% [95% Confidence Interval: 27.7–35.4] were HSV-2 positive. Rao-Scott Chi-Square analyses (seen in Table 1) demonstrated a significant difference in HIV positivity between HSV-2 positive and HSV-2 negative women: 4.8% of HSV-2 positive pregnant women were also HIV-positive, while only 0.9% of HSV-2 negative pregnant women were HIV-positive (*p* < 0.001). Similarly, HSV-2 positive pregnant women were significantly older than HSV-2 negative women (mean age of 27.3 versus 26.2). There were no significant differences in residence, education, or marital status by HSV-2 status among pregnant women.

**Table 1:** Weighted demographics of the tested women

Characteristic**	Overall		HSV-2 positive		HSV-2 negative		*P*-values
	n^a^	% (95% CI)	n	% (95% CI)	n	% (95% CI)	
Mean age	925	26.5 (26.0–27.1)	340	27.3 (26.3–28.3)	585	26.2 (25.5–26.8)	0.036*
Age category							0.031*
14–19	135	15.2 (12.7–18.0)	36	10.9 (8.1–14.5)	99	17.1 (13.7–21.2)	
20–24	245	26.6 (23.2–30.4)	88	26.8 (19.0–36.4)	157	26.6 (24.0–29.2)	
25–29	256	27.7 (22.9–33.0)	92	26.3 (19.0–35.3)	164	28.3 (23.0–34.3)	
30–34	173	18.4 (16.1–20.9)	74	22.3 (17.5–27.9)	99	16.6 (14.2–19.4)	
35+	116	12.1 (9.7–15.1)	50	13.7 (10.3–18.0)	66	11.4 (9.1–14.2)	
Residence							0.068
Urban	508	54.4 (33.6–73.9)	195	57.6 (35.6–77.0)	313	53.0 (32.4–72.6)	
Rural	417	45.6 (26.1–66.4)	147	42.4 (23.0–64.4)	270	47.0 (27.4–67.6)	
Marital status							0.484
Married or widowed	246	26.0 (21.5–31.1)	88	25.2 (18.3–33.7)	158	26.4 (22.5–30.7)	
Living with partner	487	53.3 (49.0–57.4)	173	51.0 (44.1–57.8)	314	54.3 (49.6–58.9)	
Engaged	177	18.9 (14.4–24.4)	73	21.7 (16.6–27.8)	104	17.7 (12.8–23.8)	
Single	18	1.8 (0.9–3.5)	8	2.1 (0.6–7.8)	10	1.7 (0.8–3.3)	
Education level							0.794
Primary or none	417	44.7 (35.9–53.9)	157	45.3 (33.6–57.6)	260	44.5 (36.4–52.9)	
Secondary or higher	511	55.3 (46.1–64.1)	185	54.7 (42.4–66.4)	326	55.5 (47.1–63.6)	
HIV status							<0.001*
HIV-Positive	144	2.1 (1.6–2.8)	103	4.8 (3.9–6.0)	41	0.9 (0.6–1.3)	
HIV-Negative	784	97.9 (97.2–98.4)	239	95.2 (94.0–96.1)	545	99.2 (98.7–99.4)	

^a^These prevalences exclude 3 observations where age was missing and 3 observations where residence was missing

*Significance at alpha=0.05

**This table only presents information on HSV-2 tested women, but those not tested for HSV-2 were not significantly different than those tested in terms of mean age, age group, residence, marital status, education level or HIV status

**Fig. 1:**
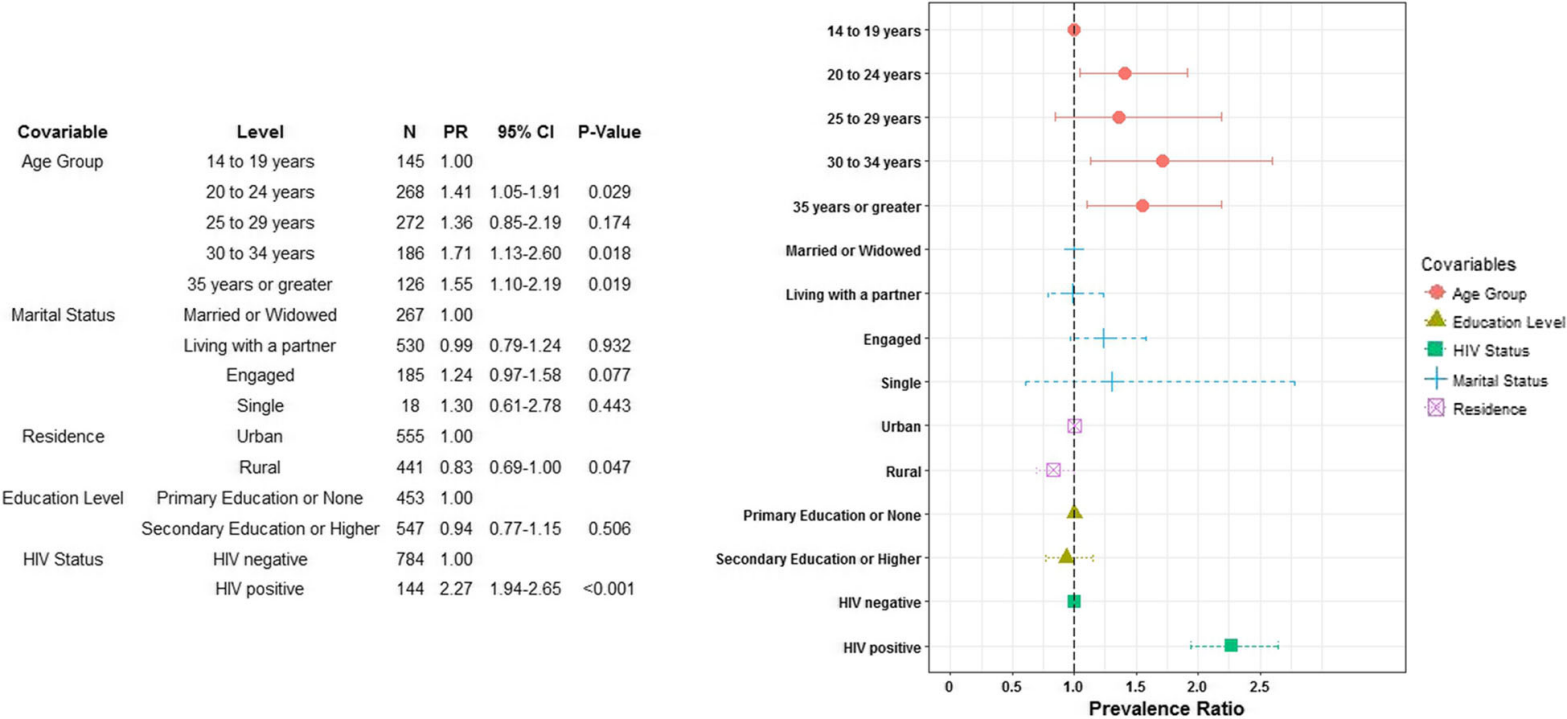
Multivariable cox-proportional hazards modeling results

Multivariable Cox proportional hazards model of HSV-2 seropositivity revealed that, when compared to those aged 14 to 19 years, those aged 20 to 24, 30 to 34, and 35 years or older all had significantly higher prevalence of HSV-2 after controlling for all other variables, as seen in Fig. 1. Those living in rural environments had significantly lower prevalence of HSV-2 when compared to those living in urban environments, after controlling for all other variables (PR: 0.83, [CI: 0.69–1.00], *p* = 0.047). HIV positivity was associated with 2.27-fold increase in prevalence of HSV-2, when compared to those who were HIV negative, after controlling for all other factors ([CI: 1.94–2.65], *p* < 0.001). Marital status and education level remained not significantly associated with HSV-2 prevalence after controlling for other variables.

The results of testing with Focus and Kalon are shown in Table 2. Five hundred and fifteen (55.5%) of the 928 tested samples had index values at or below 1.1 and were not tested with Kalon. Three hundred and forty four (37.1%) of the tested samples had an index value ≥3.5, and 70 (7.5%) had index values between 1.1 and 3.5. Nearly all of the samples with an index value ≥3.5 tested positive with Kalon (342/344, 99.4%). Of the 70 samples with values between 1.1 and 3.5, one was not further tested due to insufficient volume. Thirty-nine of the 69 tested samples (56.5%) had positive Kalon tests, 24 (34.8%) had negative Kalon tests, and six (8.7%) had indeterminate Kalon results. Among samples with an index value ≥2.5, 98.4% had positive Kalon tests.

**Table 2:** Kalon results by HerpeSelect Index

HerpeSelect Index Range	Kalon positive	Kalon negative	Kalon indeterminate	Total
	n (%)	n (%)	n (%)	n (%)
>1.1 and <1.5	1 (0.3)	12 (48)	0 (0)	13 (3.1)
≥ 1.5 and <2.0	7 (1.8)	5 (20)	3 (42.9)	15 (3.6)
≥ 2.0 and <2.5	6 (1.6)	5 (20)	1 (14.3)	12 (2.9)
≥ 2.5 and <3.0	12 (3.1)	2 (8)	1 (14.3)	15 (3.6)
≥3.0 and <3.5	13 (3.4)	0 (0)	1 (14.3)	14 (3.4)
≥ 3.5	342 (89.8)	1 (4)	1 (14.3)	344 (83.3)
Total	381 (100)	25 (100)	7 (100)	413 (100)

## Discussion

We assessed the performance of HSV-2 tests and the prevalence of HSV-2 among pregnant women in Haiti.

Despite the use of a conservative definition of HSV-2 seropositivity, the overall weighted prevalence of HSV-2 in this population was over 30%. The prevalence in these pregnant women is higher than rates reported in clients of female CSW in two departments of Haiti [20], and high compared to the prevalence in pregnant women found in India (8.7%) [25]. Our findings showed a prevalence similar to evaluations conducted among pregnant women in Wolaita zone, Ethiopia (32%) [26] but lower than those reported in some countries in sub-Saharan Africa: Munjoma et al. [27] found a prevalence of 49.1% for HSV-2 among pregnant women in Zimbabwe. Other studies have reported lower or similar prevalence of HSV-2 among pregnant women [5, 28] however difference in methods of diagnosis makes true comparison difficult between countries or regions.

The prevalence of HIV-positivity was found to be significantly higher in HSV-2 positive pregnant women than in HSV-2 negative pregnant women. In our study, the seroprevalence of HSV-2 among HIV positive pregnant women was more than two times that of women who were HIV negative after controlling for age, education level, marital status, and residence. The strong association between HSV-2 and HIV found in our study is consistent with previous research [29–32]. Studies on the direct impact of HSV-2 treatment on HIV transmission [33–35] are not conclusive, yet such findings as ours reinforce the need to continue evidence based interventions to prevent HSV-2 among young populations particularly in resource limited settings with generalized HIV epidemics.

This study found an increase in HSV-2 seroprevalence in the age groups 20–24 years, 30–34 years, and 35 years and older when compared to the 14–19 year old age group, with a peak in the 30 to 34 year old age group. This is consistent with findings from other studies which found that HSV-2 seroprevalence tends to increase with age [12, 36], although this trend was not seen uniformly across all age groups as the 25–29 year old age group, the largest one, was not significantly different to the 14–19 year old age group. This non-significance persisted even after combining the 25–29 year old age group with the 20–24 year old age group. One explanation for this lack of a uniform increase with increasing age groups may be that the tested population, which was pregnant women participating in ANC HIV surveillance, is not representative of the total population of women of reproductive age, as was reported in the other studies [12, 36].

The rates of false positive HSV-2 tests in African populations are not fully understood, but may be specific to populations of African origin and/or co-infections that are prevalent in Africa [37]. Given that the Haitian population is largely of African origin, we were uncertain whether to anticipate high rates of false positive serologic tests for HSV-2 in the population included in this study. Based on our testing, issues with performance of the Focus test do not appear to be as serious in the Haitian population as they are in some African populations. Different cutoffs could be considered for different purposes but, based on our results, defining HSV-2 seropositivity as a Focus index value of 2.5 or greater would appear to be a reasonable cutoff as agreement with the Kalon test was high (98.4%), particularly when compared to the observed agreement of 56.5% using a seropositivity definition of a Focus index value of greater than 1.1.

Our study adds to the scarce information available concerning HSV-2 infection rates and HSV-2 test performance in Haiti, in spite of some limitations. We did not conduct confirmatory testing on samples with low Focus index values (<1.1) as we opted for this analysis to consider the conservative definition for positive described elsewhere given the high proportion of false positive found at this cutoff [22, 23]. As such, we may have overlooked a problem with low sensitivity of that test; however sensitivity of the Focus test has been high across a broad array of populations. Additionally, this study was limited to a sample of women included in the antenatal surveillance effort which is not representative of all pregnant women in Haiti. As those presenting in clinics are often of higher socioeconomic status, it is possible that the true burden of disease is higher in the general population of pregnant women when compared to our sample of pregnant women seeking ANC [38]. Furthermore, we purposefully selected all HIV-positive women from the original study into this secondary analysis, as HSV-2 positivity is highly correlated with HIV-positivity, however, by doing so we may have introduced selection bias into our study [2, 4, 5]. However, HIV-positive and HIV-negative women did not differ significantly by age group, residence, education or marital status; we accounted for unequal probabilities of selection for the HIV-positive women and randomly selected HIV-negative women using sampling weights.

While we await interventions that can modify the risk of HIV acquisition and/or transmission for persons infected with HSV-2, information regarding HSV-2 rates can help inform efforts aimed at primary prevention of HSV-2 and identify populations at highest risk for HSV-2. In addition, the high prevalence found in this study population warrants development of targeted policies and strategies related to HSV-2 screening in antenatal services in spite of the absence of data on the prevalence of neonatal herpes in Haiti. The identification and the provision of adequate care and treatment to pregnant women with primary infection or recurrence of HSV-2 could reduce incidence of neonatal herpes and, among HIV-infected pregnant women, reduce the risk of vertical transmission of HIV [6, 7, 39].

## Conclusions

In this first study assessing HSV-2 infection among pregnant women in Haiti, the seroprevalence was relatively high at 31.4% and was strongly associated with HIV positivity. In light of these findings, public health interventions are warranted to increase access to HSV-2 screening in antenatal services, particularly among HIV infected pregnant women, using Focus at an index value of 2.5 or greater and to reinforce counselling on the prevention of HSV-2 acquisition in late pregnancy. With such high prevalence of HSV-2 among pregnant women, further research is needed to evaluate the prevalence and impact of neonatal herpes in Haiti in order to inform public health policies.

## Abbreviations

ANC: Antenatal care
CDC: Centers for Disease Control and Prevention
CI: Confidence Interval
CSW: Commercial Sex Worker
ELISA: Enzyme-linked immunosorbent assay
HIV: Human Immunodeficiency Virus HSV-2 Herpes Simplex Virus type 2
LNSP: Laboratoire National de Santé Publique (National Public Health Laboratory)
PMTCT: Prevention of Mother-to-Child Transmission
PR: Prevalence Ratio
